# Synthetic biology in marine cyanobacteria: Advances and challenges

**DOI:** 10.3389/fmicb.2022.994365

**Published:** 2022-09-16

**Authors:** Barbara Bourgade, Karin Stensjö

**Affiliations:** Microbial Chemistry, Department of Chemistry-Ångström Laboratory, Uppsala University, Uppsala, Sweden

**Keywords:** synthetic biology, marine, cyanobacteria, genetic tools, metabolic engineering

## Abstract

The current economic and environmental context requests an accelerating development of sustainable alternatives for the production of various target compounds. Biological processes offer viable solutions and have gained renewed interest in the recent years. For example, photosynthetic chassis organisms are particularly promising for bioprocesses, as they do not require biomass-derived carbon sources and contribute to atmospheric CO_2_ fixation, therefore supporting climate change mitigation. Marine cyanobacteria are of particular interest for biotechnology applications, thanks to their rich diversity, their robustness to environmental changes, and their metabolic capabilities with potential for therapeutics and chemicals production without requiring freshwater. The additional cyanobacterial properties, such as efficient photosynthesis, are also highly beneficial for biotechnological processes. Due to their capabilities, research efforts have developed several genetic tools for direct metabolic engineering applications. While progress toward a robust genetic toolkit is continuously achieved, further work is still needed to routinely modify these species and unlock their full potential for industrial applications. In contrast to the understudied marine cyanobacteria, genetic engineering and synthetic biology in freshwater cyanobacteria are currently more advanced with a variety of tools already optimized. This mini-review will explore the opportunities provided by marine cyanobacteria for a greener future. A short discussion will cover the advances and challenges regarding genetic engineering and synthetic biology in marine cyanobacteria, followed by a parallel with freshwater cyanobacteria and their current genetic availability to guide the prospect for marine species.

## Introduction

Modern society faces various challenges that must be addressed promptly to preserve most living organisms. The current environmental crisis is particularly pressing and threatening and demands that the present industrial and economical behaviors are entirely revised (Levi and Cullen, 2018). As such, many research, political and industrial efforts have focused on developing sustainable solutions to mitigate the climate emergency. In this context, bioprocesses, relying on living organisms for biological catalysis, stand as a key solution due to their flexibility, robustness and sustainability. Moreover, the current omics data and synthetic biology techniques available to strengthen and diversify bioprocesses further support their wider use for various industrial applications, including fuels, chemicals or pharmaceuticals production.

Of particular interest for sustainable industrial bioprocesses, cyanobacteria perform oxygenic photosynthesis, using solar energy for their metabolism while fixing atmospheric CO_2_. These prokaryotes are significant contributors to global carbon fixation in many environments, participating in ecosystem maintenance. The tremendous diversity and strain-specific variation of pigments and photosystems (Stephens et al., 2021), essential elements for photosynthesis, provide great opportunities for tailored biotechnological applications, with strains metabolically adapted to specific environmental conditions, such as high salinity, temperature and fluctuating light. To date, hundreds of cyanobacterial species have been isolated in marine and freshwater environments with new species continuously being identified and further characterized. Historically, a few model freshwater species have previously led researchers to understand cyanobacterial metabolism and photosynthetic capabilities and develop synthetic biology tools for strain engineering (Gale et al., 2019). However, marine cyanobacteria display several advantages over their freshwater counterparts, including their capacity to grow in seawater, their robustness to environmental changes and specific metabolic abilities, including biosynthesis of complex and unique biomolecules. Currently, more work is still needed to adapt many synthetic biology techniques to marine cyanobacteria and fully unlock their biotechnological and industrial potential (Khalifa et al., 2021).

This short review will briefly discuss the impressive diversity of marine cyanobacteria and the associated opportunities for unique bio-applications. The recent development of genetic tools and the advances of synthetic biology in marine cyanobacteria will be further reviewed, highlighting some of the challenges that must be addressed to move forward with these species and expand their applications. Finally, some of the more advanced synthetic biology tools developed for freshwater cyanobacteria will be summarized to draw a parallel between marine and freshwater species. While the focus of this review will be synthetic biology for industrial applications, it is worth mentioning that synthetic biology methods are also relevant for fundamental studies, such as phenotypic screening or regulation characterization, but will not be discussed here.

## Diversity, opportunities and engineering of marine cyanobacteria

### The diversity of marine cyanobacteria offers various opportunities for biotechnological applications

Marine cyanobacteria are a highly diverse group of photosynthetic prokaryotes, with relevant metabolic properties for future sustainable industrial applications. Their photoautotrophic abilities, attractive for biotechnological applications, are also incredibly important for global CO_2_ fixation to support a variety of ecosystems. In fact, some genera can fix up to four gigatons of carbon per year (Biller et al., 2015). From a biotechnological perspective, in addition to the extensive CO_2_ availability, their ability to prosper at high salinities further supports sustainability, preventing competition for drinking water. Furthermore, considering their natural habitats where temperature, nutrient availability, salinity and light intensity constantly fluctuate, marine cyanobacteria are particularly robust to environmental changes (Ludwig and Bryant, 2012), which may be beneficial in an industrial context where growth parameters might vary slightly. While most species maintain a circadian rhythm, relying on complex regulatory cascades for adaptation to fluctuating light exposure, some species, isolated from light-deprived environments, show unique adaptability to growth in darkness (Coe et al., 2021; Callieri et al., 2022), further expanding their metabolic flexibility. In fact, the diversity of marine environments has been a driving force for cyanobacterial evolution and there is a high correlation between specialized features of some species and their natural habitats (Biller et al., 2015). Considering the exceptional diversity of marine cyanobacteria, this review will not attempt to summarize each and every unique capacity previously described but will point out some species of industrial relevance and their different properties, further highlighting how this diversity can be exploited for tailored applications (Table 1). It is worth mentioning that brackish cyanobacteria, such as *Aphanizomenon flos-aquae* (Lyon-Colbert et al., 2018), will not be covered in this review but are important players in cyanobacterial biodiversity and have a significant impact on human health for bloom-forming species, as discussed elsewhere (Śliwińska-Wilczewska et al., 2019).

**TABLE 1:** Examples of genetic tools available for marine cyanobacteria and their applications for metabolic engineering.

	Species	Features	Genetic tools available	Reported metabolic engineering
**Unicellular**	*Prochlorococcus*	Unique photosynthetic apparatus Small genome Adapted to darkness	Ongoing optimisation of DNA entry (Laurenceau et al., 2020)	–
	*Synechococcus* sp. PCC 7002	Flexibility “Model” marine cyanobacterium Fast-growing	Genome integration sites (Vogel et al., 2017) Counterselection method (Begemann et al., 2013) Characterised genetic elements (Markley et al., 2015; Pérez et al., 2017; Jones et al., 2021a) Marker excision with recombinases (Jones et al., 2021b) Target downregulation (Zess et al., 2016; Gordon et al., 2016)	Nozzi et al., 2017; Hasunuma et al., 2019; Yang et al., 2021
	*Synechococcus* sp. NKBG 15041c	Fast-growing	Genome integration (Yoshino et al., 2017; Badary et al., 2018) Characteristed promoters (Badary et al., 2015)	Yoshino et al., 2017; Badary et al., 2018
	*Synechococcus* sp. PCC 11901	Fast-growing Tolerant to high temperatures and light intensities	Genome integration (Włodarczyk et al., 2020) Characterised promoters (Włodarczyk et al., 2020)	Włodarczyk et al., 2020
	*Synechocystis* sp. PCC 7338	Facultative photoautotroph	–	–
**Filamentous**	*Anabaena* sp. ATCC 33047	Nitrogen fixation	Genetic deletions (Bandyopadhyay et al., 2021)	Bandyopadhyay et al., 2021
	*Arthrospira maxima*	High protein content	–	–

Synechococcus spp. is currently the most amenable to genetic modifications. This list of examples is not exhaustive.

*Prochlorococcus* isolates, the most abundant photosynthetic organisms, have primarily been isolated from tropical and subtropical regions and have fascinated scientists for several decades, partly due to their condensed genome, with only ∼1,700 genes (Rocap et al., 2003). Within this group, strains remain highly diverse, especially regarding light adaptation, and have been reviewed in details elsewhere (Biller et al., 2015). Their photosynthetic apparatus is unique amongst cyanobacteria as it lacks the widely spread cyanobacterial phycobilisome structures and, instead, relies on divinyl chlorophyll a and b pigments in the antenna (Ting et al., 2002), a contrasting pigment composition to the phylogenetically close *Synechococcus* strains. A few *Synechococcus* species are arguably some of the most studied and well-understood marine cyanobacteria and are, in fact, almost ubiquitous to all marine environments, due to their large pigment diversity. Their fast growth rate, environmental robustness and extensive dataset further add to their industrial potential. *Prochlorococcus* and *Synechococcus* genera remain the predominant unicellular marine cyanobacteria studied currently, although selected species of other genera (Brito et al., 2017) are starting to attract interest. On the contrary, fewer filamentous species have been researched in the synthetic biology field, perhaps due to the difficulty to work around their morphology. Interestingly, some *Anabaena* species have the dual ability to fix both carbon and nitrogen and can sustain high light intensities and a wide range of temperatures and pH (Moreno et al., 2003), promising for potential industrial applications. Lastly, *Arthrospira* (also called *Spirulina*) *maxima*, another rising filamentous cyanobacterium, is gaining interest for its use as a food supplement as it offers a higher protein content than its freshwater counterpart *Arthrospira platensis*. Indeed, *Arthrospira* sp., and primarily *A. platensis*, have been extensively exploited for food, cosmetics and pharmaceutical applications. Their unique spiral morphology enables to achieve high biomass cultivations, further harvested for downstream processes (Affan et al., 2015). *A. maxima* has also recently been proposed as a suitable medium for mammalian cell cultivation (Jeong et al., 2021), therefore bypassing the need for animal-derived medium, or as a source for chlorophyll *a* extraction (Martins et al., 2021), with possible cancer therapeutics applications.

Moreover, new species are constantly isolated from various environments with highly specific metabolic properties, such as nitrogen fixation or production of complex secondary metabolites (Hoffman, 1999; Brito et al., 2017; Shiels et al., 2019). Therefore, it is very likely that additional organisms with interesting properties will be isolated in the near future. The close correlation between the natural habitat and the evolution of unique features also further promotes biotechnological applications in specific regions. Indeed, since species have adapted, for example, to extreme temperatures or different light intensities, these same species can become the workhorse of biotechnological applications in these regions, without needing further strain adaptation and accelerating the development of highly specialized applications. Moreover, it is worth mentioning that, in addition to their photoautotrophic capabilities, native secondary metabolites from marine cyanobacteria have also gained interest for their potential therapeutics applications as reviewed elsewhere (Tan and Phyo, 2020; Lopes et al., 2022). Similarly, natural pigments produced by cyanobacteria have direct applications in the food and cosmetics industries, contributing to a global greener future (Saini et al., 2018).

However, although native products remain important, the future of cyanobacteria lies in the possibility to convert them into specialized cell factories for the production of various compounds. Utilizing their complex and flexible metabolic abilities, and, in particular, solar energy and CO_2_ fixation, these applications will drive the change toward a sustainable future. This course can, however, only be achieved if routine strain engineering, mediated by robust and efficient genetic tools, is accessible for introduction and manipulation of metabolic pathways, leading to maximal compound bioproduction and strain robustness for industrial conditions. While this review focuses on the bioproduction of specific chemicals, cyanobacteria are also relevant for other biotechnological applications, such as bioremediation, which has been discussed elsewhere (Rotter et al., 2021; Touliabah et al., 2022).

### Limited genetic tools have been developed and applied in marine cyanobacteria

Genetic accessibility is crucial for chassis organisms with potential industrial applications. Indeed, this allows to engineer strains with, for example, non-native properties and improved catalytic efficiency in order to meet industrial standards, which need robust and superior strains. Numerous methods for genome editing with direct synthetic biology applications have now been established for many organisms, especially model organisms, such as *Saccharomyces cerevisiae* or *Escherichia coli*. However, the toolkit available for non-model organisms, including cyanobacteria, remains limited, preventing routine strain engineering for specific industrial aims. Expanding this toolkit is undeniably challenging, especially due to the polyploidy of most cyanobacteria (Griese et al., 2011), but vital to accelerate cyanobacterial bioprocesses.

**FIGURE 1:**
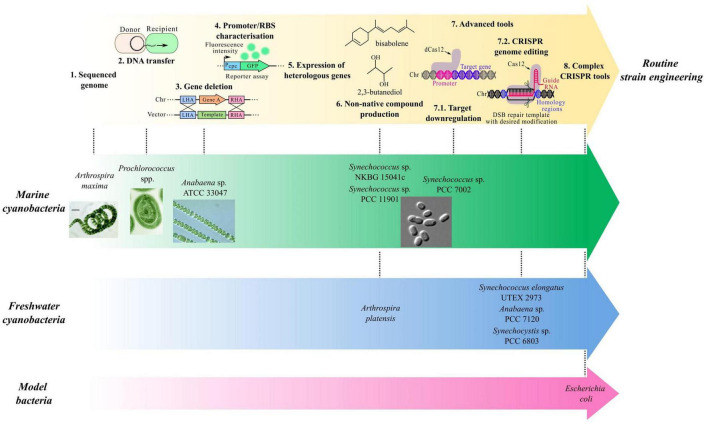
Progression of genetic tools toward routine strain engineering. Most marine cyanobacteria remain at earlier stages of tool development while freshwater cyanobacteria and model organisms have more complex genetic tools available.

Considering the importance of reliable engineering methods, several research groups have spent time and effort developing such tools (Table 1). Today, *Synechococcus* sp. PCC 7002 (hereafter *Synechococcus* 7002) is arguably the most genetically amenable marine cyanobacterium (Figure 1). In fact, several genomic integration sites have been characterized for minimal metabolic disruption, allowing insertion of genetic constructs at different loci (Ruffing et al., 2016; Vogel et al., 2017). Integration in the native plasmid pAQ1 also offers an interesting option due to the higher copy number compared to genome integration (Yang et al., 2021). Natural transformation tends to be the most common DNA transfer for genomic integration in *Synechococcus* 7002 by selecting transformants through antibiotic resistance. In addition, an acrylic acid counterselection method has previously been described where deletion of the *acsA* gene confers resistance to acrylic acid (Begemann et al., 2013). Furthermore, genetic elements are critical to achieve the required expression level. To test their activity, reporter genes, coding, for instance, for the yellow or green fluorescent protein, can be used. As such, several publications have now identified promoters with specific strength for *Synechococcus* 7002. Native promoters often are a suitable starting point as host compatibility is assured and have previously been investigated in *Synechococcus* 7002 (Ruffing et al., 2016). Additional tested promoters include a library of synthetic promoters derived from *Synechocystis* sp. PCC 6803 P*_*cpcB*_* promoter, further engineered for IPTG inducibility (Markley et al., 2015). Other inducible systems include a T7-based system (Jones et al., 2021a), a zinc-inducible promoter (Pérez et al., 2017), and a tetracycline-dependent system (Zess et al., 2016), which allow tuneable gene expression. Ribosome-binding sites also influence expression levels but are often overlooked in most chassis organisms, although a RBS library has been characterized in *Synechococcus* 7002 (Markley et al., 2015). These different genetic elements can therefore drive expression of synthetic constructs, awarding additional properties to *Synechococcus*. Importantly, a recombinase-based tool has previously been optimized for this organism to allow removal of selection markers, highly relevant for wider use of engineered strains (Jones et al., 2021b). The authors showed that both CRE and DRE recombinases were functional and compatible for recombination at their respective lox sites, allowing marker removal. Interestingly, to avoid maintaining the recombinase genes permanently in the genome, this approach exploited *Synechococcus* 7002 polyploidy where the recombinases were inserted in an essential gene, therefore unable to achieve full segregation, and maintained until marker removal, after which the wild-type locus at the essential gene took over the recombinase insertion. While these different strategies allow targeted genome-editing for expression of heterologous genes, strain stability may be limited, with accumulation of mutations and a potential decrease of productivity, as discussed elsewhere (Jones, 2014; Hitchcock et al., 2020). This limitation, although not specific to cyanobacteria, must be addressed for longer and larger cultivations in industrial plants and may be partially mediated by fine-tuned regulation of heterologous pathways. Lastly, target downregulation through RNA interference (Zess et al., 2016) and CRISPR interference (Gordon et al., 2016) by blocking target translation and transcription, respectively, has been reported and is particularly powerful for genome-wide phenotypic analysis. It can also improve target production as illustrated by increased lactate production when using CRISPR interference for elevating concentration of the lactate precursor pyruvate. While development of these tools is impressive, their direct applications for metabolic engineering are striking. In fact, a significant number of non-native target compounds have now been produced by *Synechococcus* 7002 at a laboratory scale. To name a few, bioproduction of the hydrocarbons limonene and bisabolene (Davies et al., 2014), the pigment astaxanthin (Hasunuma et al., 2019), the amino acid L-lysine (Korosh et al., 2018), the building block 2,3-butanediol (Nozzi et al., 2017), the potential aircraft fuel pinene (Yang et al., 2021), the fatty acid lauric acid (Work et al., 2015) or the biomaterial precursor polyhydroxyalkanoate (Zhang et al., 2015) has been achieved. The chemical diversity of these compounds further highlights the industrial importance of this chassis organism for a greener future. While the native metabolism offers the opportunity to synthesize complex non-native molecules, such as pigments or secondary metabolites, by providing precursors for engineered pathways, complete heterologous routes converting core metabolites, such as acetyl-CoA or pyruvate, are also relevant for target bioproduction in cyanobacteria. While this review primarily highlights synthetic biology for non-native chemical bioproduction, engineering strategies can also improve the biosynthesis of native secondary metabolites, such as antioxidants or UV protectants (Vega et al., 2020), as discussed extensively elsewhere (Jeong et al., 2020). As such, cyanobacterial target bioproduction is suitable for diverse groups of chemicals. Further more additional metabolic engineering reported in these studies, such as deletion of the glycogen synthase (Davies et al., 2014), further illustrate that our current understanding of the metabolism can support metabolic manipulation and strain optimization. These strategies can now also be guided by *in silico* methods, such as a genome-scale method, available for *Synechococcus* 7002 (Hendry et al., 2016), allowing more informed designs.

*Synechococcus* 7002 remains undeniably the most genetically accessible marine cyanobacterium. However, encouragingly, some similar tools have been developed for *Synechococcus* sp. NKBG 15041c, achieving bioproduction of glycogen (Badary et al., 2018) and omega-3 fatty acids (Yoshino et al., 2017) through metabolic engineering. Further promoter development and characterization is also underway (Badary et al., 2015), therefore expanding the genetic toolkit available for this strain. The newly discovered strain *Synechococcus* sp. PCC 11901 has also been modified through natural transformation for free fatty acids production, with rapid tool development after isolation (Włodarczyk et al., 2020). On the other hand, *Prochlorococcus* strains, although phylogenetically close to *Synechococcus* spp. (Biller et al., 2015), remain highly resistant to genetic modifications. Despite an extensive dataset on its physiology and genomics (Berube et al., 2018), DNA transfer into *Prochlorococcus* seems particularly challenging as discussed elsewhere (Laurenceau et al., 2020). This further highlights the difficulty to adapt genetic tools to non-model species of any phylum, which requires to develop a robust DNA transfer method that must consider any species-specific biological and physiological obstacles, such as restriction-modification systems (Johnston et al., 2019), membrane composition or requirements for axenic cultivation.

Furthermore, the filamentous cyanobacterium *Anabaena* sp. ATCC 33047 has yet to be engineered for heterologous gene expression but deletion of *nblA*, coding for a small protein involved in phycobilisome degradation, has recently been reported through homologous recombination. This work required to create a specific helper strain that carried *Anabaena* sp. ATCC 33047 methylase genes to allow successful conjugation (Bandyopadhyay et al., 2021). This first report of genetic modifications in this strain will most likely lead to additional strain engineering, which can be guided by this host’s genome-scale model (Hendry et al., 2021).

Lastly, an impressive progress toward a robust genetic toolkit for *Arthrospira platensis* has recently been reported (Jester et al., 2022), further discussed later in this review. Earlier phylogenetic analyses suggested that *A. platensis* and *A. maxima* were closely related (Singh and Dhar, 2011), suggesting that these new tools could be transferred to *A. maxima.* However, genomics datasets have only recently been acquired for *Arthrospira* sp. and it is, therefore, premature to conclude how these two species differ. Nonetheless, a recent comparative genomic analysis (Misztak et al., 2021) proposed that the two species are, in fact, in two different *Arthrospira* groups, thus questioning whether tool transferability could be achieved in these two hosts.

## The genetic toolkit for freshwater cyanobacteria is more extensive

Although great progress on toolkit development has been made in some marine cyanobacteria, freshwater species are slightly more advanced in terms of complex genetic tools (Figure 1). For example, many promoters, including native, heterologous and inducible systems (Englund et al., 2016; Wang et al., 2018; Behle et al., 2020), have now been characterized in *Synechocystis* sp. PCC 6803 or *Synechococcus elongatus* PCC 7942 (Berla et al., 2013; Sengupta et al., 2020). This allows tuneable target expression, essential for pathway expression and metabolic engineering.

In addition, genome editing is still routinely achieved through homologous recombination in most cyanobacteria, for which neutral integration sites have been identified (Almeida et al., 2017; Nagy et al., 2021). Counter-selection systems have been developed for transformant selection, such as *sacB* for sucrose sensitivity (Lagarde et al., 2000) or the protein synthesis inhibitor *mazF* (Cheah et al., 2013) in *Synechocystis* sp. PCC 6803, leading to markerless strains, important for biosafety in an industrial context. This has recently been further expanded with the identification of additional counter-selection methods in *Synechococcus elongatus* UTEX 2973 (Chen et al., 2021). Jones et al. (2021b) also showed that their CRE/DRE recombinase systems were functional in *Synechocystis* sp. PCC 6803.

Although these counter-selectable methods have shown great potential, they remain particularly time-consuming in polyploid organisms, which require multiple segregation steps to isolate pure mutants. This limitation also complicates genomic modifications at multiple sites. CRISPR (Clustered Regularly Interspaced Palindromic Repeats)-Cas tools have revolutionized genome editing in various organisms, allowing rapid and markerless genomic modifications. This RNA-guided method, reviewed elsewhere (Zhang and Huang, 2022), causes a double-stranded break, which must be repaired for cell survival. The repair mechanism can be harnessed to insert desired modifications. Genome editing with CRISPR has now been performed in *Synechocystis* sp. PCC 6803, *Synechococcus elongatus* PCC 7942, *Synechococcus elongatus* UTEX 2973 and *Anabaena* sp. PCC 7120 (Li et al., 2016; Ungerer and Pakrasi, 2016; Lu et al., 2022), a tremendous step toward a robust and modern genetic toolkit for cyanobacteria. In addition, multiplexing (i.e., simultaneous targeting of several genes) of CRISPR interference has been reported in freshwater cyanobacteria (Yao et al., 2016; Santos et al., 2021) but still lacking in marine species, preventing the use of CRISPRi in systemic studies.

Some of the reported tools have been adapted in both unicellular and filamentous species, suggesting that morphology is not impeding tool development. In fact, a previous CRISPR tool showed transferability in phylogenetically distinct unicellular and filamentous species (Ungerer and Pakrasi, 2016). In another study, the neutral site 2 from *S. elongatus* PCC 7942 was introduced in *Anabaena* sp. PCC 7120 genome for direct tool transferability without requiring additional cloning steps (Taton et al., 2020), allowing introduction of a cryptomaldamide gene cluster from a marine cyanobacterium in both species. Interestingly, cryptomaldamide was only detected in *Anabaena*, hypothesized to be more suitable for production of complex secondary metabolites. This further illustrates that pathway transferability between unicellular and filamentous species might not be as straight-forward as tool transferability, likely due to metabolic differences. In addition, tools developed specifically for filamentous species, such as *Arthrospira platensis*, have also been reported. In fact, optimization of the genetic engineering techniques led to the development of a potential oral therapy for *Campylobacter* infections (Jester et al., 2022), harnessing the edible properties of spirulina.

Finally, an important tool for routine and rapid genetic engineering is shuttle vectors, able to replicate within the cyanobacterial population. This prevents time-consuming genomic integration for genetic element and tool characterization and provides higher expression levels. The broad-host range replicon RSF1010 has been used widely in freshwater cyanobacteria (Mermet-Bouvier et al., 1993; Taton et al., 2014) and additional shuttle vectors, based on native plasmids, have recently been engineered for *Synechocystis* sp. PCC 6803 (Jin et al., 2018; Liu and Pakrasi, 2018). Although beneficial for many applications, strains carrying shuttle vectors tend to have an increased instability, which can be problematic in an industrial context. Shuttle vectors are still missing for most marine cyanobacteria, therefore preventing rapid tool characterization before moving to strain engineering. This requires to identify compatible origins of replication capable of maintaining a shuttle vector within the host population. Broad host-range origins or origins derived from the host’s native plasmids may be interesting to explore initially for potential compatibility, as achieved in some freshwater species.

## Discussion and outlook

The potential of marine cyanobacteria for various biotechnological applications and, more importantly, sustainable compound bioproduction is remarkable. The diversity of these species and their unique metabolic properties, evolved to survive in changing environments, offer tailored possibilities for industrial bioprocesses, such as large-scale photobioreactors for target bioproduction. To fully unlock their potential, marine cyanobacteria must be genetically accessible to further engineer strains for industrial purposes. While some progress has already been made, the current genetic toolkit remains insufficient. In fact, a clear discrepancy persists between species where some *Synechococcus* species have been and are still leading the field. On the other hand, despite the current knowledge and techniques available on genetic systems, some species continue to resist genetic modifications, as exemplified by the studied *Prochlorococcus*. While some biological challenges must participate in this discrepancy, additional aspects, such as historical attention and devoted resources, further contribute to the uneven genetic accessibility of marine cyanobacteria.

Moreover, the genetic toolkit for freshwater cyanobacteria is undeniably more extensive, with complex tools developed for several genera. Considering how phylogenetically close some marine and freshwater species are, the transferability of methods is highly probable. In fact, a proteomic analysis of *Synechocystis* sp. PCC 6803 and PCC 7338 showed how close species can be, although protein regulation reflected their respective natural habitat (Kwon et al., 2020). Similarly, genetic elements from freshwater species have shown to be functional in marine cyanobacteria (Markley et al., 2015), suggesting possible transferability. As such, adaption of genetic techniques similar to freshwater species is hopeful, especially in a growing context for the search for bioprocesses. In fact, the available research on marine cyanobacteria does not suggest that challenges specific to these species prevent their genetic engineering. Instead, the current lack of tools reflects that these species have been historically less explored than their freshwater counterparts. In addition, their extreme diversity prevents from addressing these obstacles globally as it would be inappropriate to assume one strategy would succeed in all species. Instead, strain-specific methods will be needed for efficient genetic manipulation. To achieve this, optimized growth conditions and robust methods for foreign DNA introduction are key first steps to build on for more complex genetic tools. This, of course, might prove difficult for interesting chassis organisms, such as *Prochlorococcus*, for which engineering obstacles remain unclear (Laurenceau et al., 2020).

However, it is worth pointing out that, despite the discussed advances in freshwater species, cyanobacteria in general remain behind in terms of complex genetic tools compared to model prokaryotes, such as *Escherichia coli*, for which, for example, sophisticated CRISPR tools, such as prime-editing (Tong et al., 2021), have been developed. Although adapting genetic tools to any organism is difficult, the polyploidy of cyanobacteria is particularly challenging to address and modulate and significantly contributes to limiting tool development. In particular, the lack of understanding of chromosomal copy fluctuations and their related environmental clues prevent to overcome these issues systematically. As such, further research efforts are needed to complete the genetic toolset for both marine and freshwater cyanobacteria for their unlimited industrial use.

## Author contributions

BB: conceptualization, initial writing, and manuscript revision. KS: conceptualization and manuscript revision. Both authors contributed to the article and approved the submitted version.
